# Public attitudes towards obesity policies on the island of Ireland; exploring the relationship with biopsychosocial characteristics

**DOI:** 10.1186/s12889-025-24216-8

**Published:** 2025-10-09

**Authors:** Eleni Spyreli, Emily Heery, Aoibhin Kelly, Mirjam M. Heinen, Laura McGowan

**Affiliations:** 1 Centre for Public Health, School of Medicine, Dentistry and Biomedical Science, Queen’s University Belfast, Belfast, UK; 2 Library and Research Service, Houses of the Oireachtas Service, Oireachtas, Dublin, Ireland; 3 National Nutrition Surveillance Centre, University College Dublin, Dublin, Ireland

**Keywords:** Obesity prevention, Attitudes towards policy, Island of ireland, Biopsychosocial characteristics

## Abstract

**Background:**

Public attitudes towards policies targeting obesity can influence their implementation. Gaining a better understanding of the levels of public agreement with different policies and their determinants can contribute to the development of effective approaches that can halt the rise of obesity. This study aimed to: (1) group a number of different regulatory approaches to preventing obesity into coherent categories; and (2) explore how anthropometrics (bio), health behaviours and obesity-related beliefs and experiences (psycho) and social determinants (social) may influence agreement with different obesity-related policies on the island of Ireland.

**Methods:**

A cross-sectional telephone survey collected data from a randomly selected quota sample of 1000 adults who live in the Republic of Ireland and Northern Ireland. Participants reported their level of agreement with 39 policies considered to impact upon obesity risk. They also provided information on biopsychosocial factors including demographics, anthropometrics, diet and physical activity behaviours, obesity-related beliefs and experiences of weight discrimination. Principal Component Analysis (PCA) was performed to organise the policies into coherent groups. Multiple linear regression examined the presence of a relationship between attitudes towards a class of policies and other collected variables.

**Results:**

Five policy scales including thirty-three policies were obtained: (1) *Enabling healthier environments and communities*, (2) *‘Hard’ polices*, (3) *Resources*, (4) *Promotion and information on health and food* and (5) *Advertising restrictions on unhealthy food*. Higher levels of agreement with all policy categories was associated with: older age; living comfortably on current income; being physically active; and endorsing health risks of obesity. Women reported greater agreement with *Enabling healthier environments*, *‘Hard’ polices* and more *Resources*, compared with men. Respondents who viewed maintaining a healthy weight as costly were less likely to agree with *Resources*, *Promotion and information*, and *Advertising restrictions*. Participants with personal experience of weight discrimination were less likely to agree with *‘Hard’ policies*.

**Conclusion:**

This study reveals a considerable variation in public attitudes towards obesity policies which varied by individual biopsychosocial characteristics. Present findings have important implications for policy makers tasked with designing and implementing acceptable approaches to prevent obesity at a population level.

**Supplementary Information:**

The online version contains supplementary material available at 10.1186/s12889-025-24216-8.

## Background

Obesity is a complex, chronic disease in which excess adiposity impairs health [1]. In 2019, an estimated five million deaths globally were attributed to obesity, according to The Lancet [2]. Living with excess adiposity in adulthood increases the risk of developing type 2 diabetes, cardiovascular disease, certain types of cancer and chronic respiratory and gastrointestinal disorders [3]. In childhood and adolescence, overweight is a predictor of obesity in adulthood and early onset of disease [4]. Beyond physical health, the psychological and social impact of obesity is becoming better understood. Living with excess adiposity can be highly stigmatising, with many children facing weight-based discrimination and bullying that can hinder their academic performance and mental wellbeing [5]. Similarly, adults living with obesity often face negative beliefs in everyday life, including from their healthcare providers, leading some to avoid seeking healthcare [6].

The health and social consequences of living with excess adiposity are particularly concerning considering the rising global obesity rates [7]. Similar trends are observed on the island of Ireland [8], which comprises two countries, the Republic of Ireland (RoI) and Northern Ireland (NI); with the latter being part of the United Kingdom (UK). According to the latest national surveys, one in five adults in RoI and one in four in NI are living with obesity [9, 10]. Childhood obesity is also prevalent, affecting one in four children in both countries [10, 11]. These rates are marked by widening inequalities with children in the most deprived areas experiencing almost a two-fold increase in risk of developing obesity compared to those from the least deprived areas [12].

In response to the rising prevalence of obesity on the island of Ireland, the Departments of Health in the two jurisdictions introduced their national action plans. In 2016, RoI published “*A Healthy Weight for Ireland”*, an action plan aimed at increasing the number of people with a healthy weight by 2025 [13]. The plan acknowledged that there are multiple determinants of developing excess adiposity that may impair health, which include the environment, genetic makeup, access to healthy and affordable food, exercise, culture and education. Similarly, NI introduced “*A Fitter Future for All”* in 2012, a strategic framework which aimed to create an environment that reduces the risk of developing overweight in the population and promotes the adoption of healthier behaviours with specific preventative strategies targeting the distinct stages of life [14]. The effectiveness of these interventions currently is being monitored through regular national surveys assessing diet, physical activity and body mass index (BMI) in both countries [15, 16].

At the same time, the evidence base on effective approaches to tackling obesity is growing and the value of whole-system approach (WSA) is being increasingly highlighted. WSAs consider the multifactorial drivers of overweight and obesity and employ a range of comprehensive initiatives that target different levels of our environment (e.g., industry, food environments and availability, legislation, social beliefs) and different stages of life [17]. These initiatives have the potential to achieve greater systemic changes within a population, compared to traditional single-strategy approaches.

Attention has also been drawn to the attitudes of the public towards the suggested measures. As highlighted by WHO, public acceptability is a key consideration for policy makers, as it can influence the development, implementation and success of health-related policies [18]. Acknowledging the link between public agreement with policies and their success, numerous surveys have explored what might drive acceptability of obesity-related strategies and regulations [19–25]. These studies have looked at several potential determinants of agreement with obesity-prevention strategies including sociodemographic characteristics (age, gender, education, ethnicity), causal attributions of overweight and obesity, even political ideologies. Yet, most research to date has focused on a limited set of policies or on a specific type of policies (e.g. childhood-specific strategies). More evidence from nationally representative surveys is needed to better understand public attitudes towards a broad range obesity-related interventions that form part of a WSA and address various aspects of the current environment and stages across the lifespan.

This study aimed to: (1) cluster a number of different regulatory approaches to preventing obesity into coherent categories; and (2) examine the relationship between various biopsychosocial-related characteristics and the level of agreement with obesity prevention approaches. Findings of this exploration can help inform the design of, and advocacy for appropriate and targeted public health communications and interventions to support the population to maintain a healthy weight.

## Methods

### Ethics approval

A cross-sectional telephone survey arose as part of a broader study exploring public acceptability of policies for the prevention of obesity on the island of Ireland [26]. The study was approved by the Health Research Ethics Committee of University College Dublin, with a supplementary approval obtained from the Research Ethics Committee of Faculty of Medicine, Health & Life Sciences, Queen’s University Belfast. All participants were briefed in advance on what the interview will entail and provided oral consent in the preamble of the interview.

### Participant recruitment

Participant selection and data collection was coordinated by a market research agency, *Social Market Research*. Eligible participants were adults who lived in NI and in RoI and were able to provide informed consent. Recruitment aimed for a sample of 1000 adults and utilised a random quota sampling approach, whereby quotas were set for each demographic (age, gender and socioeconomic group) in each area.

### Survey design and data collection

Data collection was conducted in October and November 2020 using a telephone-based Computer Assisted Personal Interviewing. A unique identifier was allocated to each participant. The interviews were recorded while maintaining anonymisation and the recordings were kept for 90 days, after which they were destroyed.

The survey was pilot tested for length and suitability in two stages with 30 people before it was finalised. The interviews lasted for approximately 20 min. The questions included in the interview can be found in Supplementary Fig. 1. Participants were invited to state their level of agreement with 39 obesity-related policies, which were identified following a comprehensive literature review and bult upon previous research on the island of Ireland [26, 27]. Their answers were recorded for each policy on a 5-point Likert scale ranging from *Strongly agree* to *Strongly disagree*. The survey also captured information on other characteristics in accordance with the biopsychosocial model, which suggests that a combination of biological, psychological (beliefs, behaviours), and social factors (environment, culture) impact on health [28]. These characteristics included demographics (age, gender, education, occupation, location of residence), self-perceived ability to cope financially, self-reported weight and height, self-perceived health, diet and physical activity, views on the outcomes of living with obesity and personal experiences of weight-based discrimination.

### Statistical analysis

Two policy items relating to health insurance were only applicable to RoI and therefore, were removed from the analysis. This is due to differences between the healthcare systems in the two countries. In NI, all residents are entitled to access health services at no cost through the National Health System (NHS), whereas RoI has a largely privatised health insurance system [29]. Following the removal of these two items, the analysis was conducted with 37 obesity policies relevant to the population of the entire island of Ireland.

### Principal component analysis

An exploratory Principal Component Analysis (PCA) was conducted on the 37 policies. The aim was to reduce the complexity in our dataset by grouping related policies and identifying underlying patterns to aid data interpretation [30]. Previous explorations have also used PCA as an approach to organising data in relation to attitudes towards obesity policies [20–22, 24].

Components were extracted based on Eigenvalue, Scree plot and their interpretability i.e., whether they formed coherent groups of policies. An Oblimin rotation with Kaiser Normalisation was selected assuming that the components are correlated to one another [31]. As per relevant literature [32, 33], the items/policies that did not load sufficiently to any components (i.e. loading coefficient < 0.4) were removed one by one: removing first the item/policy that displayed the smallest loading coefficient and repeating the PCA without this item; this process was followed until all policies in the pattern matrix loaded sufficiently. The Cronbach’s *α* coefficient was used to assess whether the components derived from the PCA could form coherent scales; a value of 0.7 was set as the minimum acceptable value for internal consistency [32].

Additional robustness checks were performed including: a bootstrapping procedure with 1,000 samples calculating confidence intervals (95%) for each loading and eigenvalue; a split-sample replication reporting Tucker’s congruence coefficient; and a parallel analysis. Robustness analysis was performed using R (version 4.5.1).

### Regression analysis

The scales obtained from the PCA were used to create the outcome variables. These variables took values that were calculated as follows: obtaining the average score by summing the values of level of agreement with every policy (1–5) and by dividing them by the number of valid cases in each policy group. This process was undertaken for the cases where there was sufficient data (≥ 50% of the number of policy items grouped under each scale); for the cases that did not meet this condition, the average score values were reported as missing. Linearity was assessed for every scale through a histogram and the skewness/kurtosis statistics, based on which the normal distribution was established.

The covariates included:

- Demographics: gender, age, socioeconomic status (determined by the occupation of the highest income earner in participant’s household), education, location of residence.
- Self-reported weight and height status was used to calculate body mass index (BMI).
- Self-perceived ability to cope financially.
- Beliefs regarding the consequences of obesity, as measured by the validated Obesity Beliefs Scale including: Health Beliefs, Social & Aesthetics Beliefs and Cost Beliefs [34].
- Past personal experiences of weight stigma, i.e. of bias and/or discrimination and/or social exclusion due to one’s body weight.
- Self-perceived diet and physical activity level.
- Self-perceived health.

Univariate analyses were performed to test the presence of correlations between each covariate and the outcome variable i.e. each one of the five policy scales. This was done through a linear regression analysis that adjusted for age and gender. Covariates with a *P* value < 0.1 in the univariate model were considered statistically significant to allow this covariate to be included in a final multivariate model.

Multiple linear regression was performed with each one of the policy scales as the outcome variable; a total of five multivariable regressions were performed. All variables highlighted as significantly correlated in previous univariable analysis were included as independent variables in the multivariable model. Dummy variables were created for all categorical variables that were included in the regression model: education; BMI category; ability to cope financially; self-perceived health; dietary quality; and physical activity. *P* values < 0.05 were considered statistically significant.

PCA and regression analyses were performed using IBM SPSS Statistics 27.

## Results

### Principal component analysis

Items loaded significantly onto five components accounting for 63% of total variance. The five categories of obesity policies were labelled as follows:

1. Policies that enable people to engage in health-promoting behaviours: “*Policies that enable healthier environments and communities*”, (Cronbach’s alpha = 0.91).
2. Laws and regulations which can limit choice and introduce financial incentives, subsidies and taxes relating to the adoption of healthy diet and physical activity behaviours: “*‘Hard’ polices*”, as per the definition by Banerjee et al. [35] (Cronbach’s alpha = 0.86).
3. Policies that allowed for additional resources to promote health in the population: “*Resources*”, (Cronbach’s alpha = 0.69, which, when rounded, meets the accepted threshold of 0.7).
4. Promotional campaigns around healthy eating and nutritional labelling: “*Promotion – information on health and food*”, (Cronbach’s alpha = 0.85).
5. Advertising restrictions: “*Advertising restrictions on unhealthy food*”, (Cronbach’s alpha = 0.89).

The following four items/policies did not load sufficiently to any components and were therefore removed from the final PCA and excluded from subsequent analysis: (1) Value added tax rates should be lower for healthy foods and higher for unhealthy foods; (2) The government should impose limits on certain ingredients on food companies to improve the nutritional content of processed foods; (3) Vending machines selling unhealthy food should be banned from our schools; (4) The government should provide resources to improve exercise and playground facilities.

Results of the PCA robustness checks indicated strong loadings for most variables/policies, good replicability of the PCA structure across re-samples and confirmed the robustness of a 5-component solution (Supplementary Tables 2–4).

The descriptive statistics of each scale/category of policies can be found in Table 1. The resulting 33 items included in the five final categories of obesity policies along with their loading factors can be seen in Table 2. The list of policies and level of agreement (%) with each policy can be seen in Supplementary Table 1.

**Table 1:** Descriptive statistics for the five final categories of obesity policies: average scale scores, standard deviation, range and cronbach’s alpha coefficient

	Mean ± SD	Min – Max	Cronbach’s α
Enabling healthier environments and communities (*N* = 1042)	4.37 ± 0.55	1.44–5.00	0.91
‘Hard’ policies ^1^ (*N* = 1049)	3.73 ± 0.71	1.00–5.00	0.86
Resources (*N* = 1045)	3.93 ± 0.62	1.00–5.00	0.69 *
Promotion – Information on health and food (*N* = 1049)	4.01 ± 0.64	1.00–5.00	0.85
Advertising restrictions on unhealthy food (*N* = 1049)	3.88 ± 0.78	1.00–5.00	0.89

* The Cronbach’s α for this category,* when rounded*,* meets the threshold of 0.7 so was accepted as sufficient.*

^*1*^ *‘Hard’ policies are laws and regulations that can limit choice and introduce financial incentives*,* subsidies and taxes relating to the adoption of healthy diet and physical activity behaviours* [35]

**Table 2:** Final principal components analysis pattern matrix

	Component				
	1	2	3	4	5
*Enabling healthier environments and communities*					
The government should make sure that primary school meals meet a healthy standard of nutrition	0.850	−0.017	0.048	−0.060	0.169
The government should make sure that secondary school meals meet a healthy standard of nutrition	0.843	−0.022	0.084	−0.006	0.177
The government should make sure that meals available in hospitals meet a healthy standard of nutrition	0.841	−0.009	−0.038	−0.029	0.086
The government should make sure that meals provided at workplaces meet a healthy standard of nutrition	0.717	0.085	0.136	0.011	0.102
The government should work with the food companies to improve the nutritional content of processed foods	0.577	0.176	0.018	0.121	0.093
Children should have to participate in a minimum of 30 min exercise a day while at school.	0.539	0.359	−0.082	0.235	−0.303
Education to promote healthy eating should be provided in all schools.	0.502	−0.176	0.099	0.458	0.102
Practical education in food preparation should be taught in all schools	0.501	−0.220	0.070	0.449	0.089
The government should subsidise fruit and vegetables to promote healthier eating.	0.498	0.113	0.380	0.008	−0.233
*‘Hard’ policies* ^*1*^					
Portion sizes in restaurants and fast-food shops should be restricted	−0.093	0.749	0.151	−0.118	0.115
There should be a ban on sales promotion and special offers on unhealthy foods	−0.080	0.730	0.084	0.046	0.204
The government should extend the sugar drinks tax to include all sugary foods to promote healthier eating	0.156	0.623	−0.029	0.030	0.111
Children’s height and weight should be routinely measured to monitor rates of growth in the population	0.150	0.619	−0.356	0.184	0.069
There should be planning regulations to restrict the development of certain food outlets in towns and cities	−0.154	0.609	0.379	0.027	0.107
There should be planning regulations to restrict the development of fast-food outlets in areas nearby to schools	−0.109	0.537	0.341	0.143	0.066
There should be a tax incentive to encourage sports participation with a tax break for purchasing sports equipment	0.291	0.504	−0.045	0.196	−0.014
The government should impose taxes on unhealthy foods and use the proceeds to promote healthier eating	0.313	0.468	−0.007	0.110	−0.050
The government should try and make cities so that people are encouraged to be more active and healthier	0.361	0.447	−0.172	0.116	0.128
*Resources*					
The government should provide vouchers to low-income families to buy healthy foods at reduced prices	0.357	−0.042	0.630	−0.076	−0.078
The government should provide resources to encourage women to breastfeed	−0.128	0.219	0.588	−0.002	0.164
There should be measures e.g. free delivery to support easy access to healthy food for elderly or those on low income	0.264	−0.049	0.514	0.179	0.121
TV-stations should give free air-time to governmental campaigns that promote healthier eating	0.098	0.059	0.433	0.425	0.085
*Promotion – information on health and food*					
The food industry should help pay for governmental campaigns that promote healthy eating	−0.163	0.018	−0.070	0.874	−0.026
All restaurants should be required to provide calorie and nutrient information on menus	−0.015	0.058	−0.080	0.794	0.149
All foods should be required to carry understandable labels with calorie and nutrient information	0.082	0.068	0.038	0.682	0.198
The government should spend money on effective campaigns informing people about the risks of unhealthy eating	0.277	−0.151	0.123	0.506	0.286
The government should reward companies for healthy food innovations	0.066	0.350	0.305	0.454	−0.307
The government should subsidise businesses which provide programmes to support their employees in healthy eating	0.119	0.166	0.274	0.432	0.054
*Advertising restrictions on unhealthy food*					
The government should restrict advertising for unhealthy food in public spaces (bus stops, trains stations, hospitals)	0.122	0.248	0.065	0.022	0.689
The government should restrict advertising for unhealthy food aimed at children on the internet (apps, social media)	0.115	0.232	−0.010	0.060	0.676
The government should ban advertising for unhealthy food that is aimed at children	0.102	−0.018	0.098	0.278	0.629
The government should ban advertising for unhealthy food that is aimed at adults	0.132	0.129	−0.051	0.273	0.538
The government should ban companies of unhealthy foods from sponsoring children organisations, events, sport teams	0.163	0.415	0.081	−0.006	0.479

*The threshold set for the strength with which an item significantly loaded on a component was ≥ 0.4*

^*1*^ *‘Hard’ policies are laws and regulations that can limit choice and introduce financial incentives*,* subsidies and taxes relating to the adoption of healthy diet and physical activity behaviours* [35]

### Participant characteristics

A total of 1049 adults provided data that were included in the final analysis. The average age was 47 years (range = 18–99). There was almost an equal split between men and women and those of higher and lower socioeconomic status, in accordance with the sample quotas set out from the study design stage. Additionally, 30% of responders did not self-report their weight or/and height during the telephone survey and therefore a BMI value could not be calculated for them; they were designated as a separate category entitled ‘non-responders’. Table 3 provides more information on participants’ characteristics.

**Table 3:** Participant characteristics

**Continuous variables**	**Median**	**Q3-Q1**	**Min - Max**
Age, y (*N*=1049)	46	30	18 - 99
Health Beliefs Subscales (*N*=1046) *	20	4	6 - 28
Social & Aesthetics Beliefs Subscale (*N*=1039) *	15	4	4 - 20
Cost Beliefs Subscale (*N*=1049) *	14	5	5 - 25

Categorical variables	Number		%
Location of residence (*N*=1049)			
RoI / NI	727 / 322		69.3 / 30.7
Gender (*N*=1045)			
Women / men	549 / 496		52.5 / 47.5
Education (*N*=1046)			
University (UG or PG degree)	217		20.7
Secondary/further education (e.g.NVQ)	615		58.6
Below secondary/compulsory level	213		20.3
Socioeconomic status (*N*=1049)			
ABC1 / C2DE	450 / 599		42.9 / 57.1
Ability to cope financially			
Comfortable	358		36.7
Coping	373		38.3
Difficult	243		25.0
Weight status (*N*=1049)			
Healthy weight	363		34.6
Living with overweight	231		22.0
Living with obesity	132		12.6
Non responders	321		30.6
Self-perceived overall health (*N*=1049)			
Good health / Not good health	733 / 316		69.9 / 30.1
Self-perceived diet quality (*N*=1049)			
Eating healthy / Not healthy	816 / 233		77.8 / 22.2
Self-perceived physical activity (*N*=1049)			
Active / Not active	714 / 335		68.0 / 32.0
Experience of weight stigma (*N*=1049)			
Yes / No	109 / 940		10.4 / 89.6

*Abbreviations*: *y* years, *RoI* Republic of Ireland, *NI* Northern Ireland, *UG* undergraduate, *PG* postgraduate, *NVQ* national vocational qualification

* Subscales of the Obesity Beliefs Scale, from Swift et al. [34]

### Associations with demographics, anthropometrics, health behaviours and obesity-related beliefs and experiences

In terms of *policies that enable healthier environments and communities*, women, older age, living with obesity, engaging in physical activity and supporting the negative health consequences of obesity were significantly associated with higher scores of agreement with these policies. On the other hand, not providing/knowing one’s height and weight (BMI non-responders), finding it difficult to cope/just coping financially and believing that obesity comes with negative social and aesthetic consequences were inversely associated with levels of agreement with *policies that enable healthier environments*.

The multivariable regression model examining potential predictors of agreement with *‘hard’ policies* showed a direct relationship with women, of older age, not providing a BMI, eating healthy, being physically active and believing in negative health and social outcomes of obesity. However, living in RoI, finding it difficult to cope financially and personal experience of weight-based stigmatisation showed an inverse significant relationship with scores of agreement with *‘hard’ policies*, when compared with living in NI, living comfortably on current salary and not having experienced weight stigma respectively.

The results from the regression analysis focusing on the level of agreement with *more resources* showed that the following groups exhibited greater acceptability of these policies: women compared to men; older individuals; those living with obesity compared to those living with healthy weight; those who reported being physically active; those reporting good overall health; and those who believe that obesity has negative health outcomes. Conversely, individuals living in RoI and those who held stronger beliefs that achieving a healthy weight is attained at a high cost/difficult scored lower in relation to agreement with these policies.

In the regression model that looked at the relationship between attitudes towards *promotion- and information-related policies* and the collected variables, age was significantly associated with increased levels of agreement with these policies. Similarly, individuals who were physically active were likely to have higher agreement scores, compared with those who did not perceive themselves as active. Furthermore, a higher score in the obesity health beliefs scale (i.e. greater endorsement of the health benefits of living with a healthy weight) was associated with an increase in agreement with policies related to *promotion and information on health and food*. Individuals who found it difficult to cope financially and those who believed that maintaining a healthy weight is costly/difficult had lower levels of agreement with these policies.

Finally, in this sample, older participants, those who did not complete secondary education, who reported healthy eating, reported being physically active and believed that obesity has negative health outcomes demonstrated higher levels of agreements with *advertising restrictions*. On the other hand, those with lower levels of agreement with this group of policies lived in RoI, from a lower socioeconomic grouping, found it difficult to cope financially, believed that obesity has negative consequences on one’s social life and that maintaining a healthy body weight is costly/difficult. All the above associations and the results from the regression analyses can be found in Table 4.

**Table 4:** Results from regression analyses with outcome variables being attitudes towards the 5 groups of obesity policies

		**Policies enabling healthier environments and communities**	**‘Hard’ policies** ^**1**^	**Resources **	**Promotion – information on health and food**	**Advertising restrictions on unhealthy food**
		Unstandardised Coefficient Beta (95% Confidence Intervals)				
Location	NI	*	Ref	Ref	*	Ref
	RoI		-0.276^3^ (- 0.365 - -0.186)	-0.139^2^ (-0.227 - -0.052)		-0.227 ^3^ (-0.333 - -0.121)
Gender	Men	Ref	Ref	Ref	Ref	Ref
	Women	0.062 ^1^ (0.006-0.118)	0.087 ^1^ (0.009-0.165)	0.110 ^2^ (0.032-0.187)	0.023 (-0.052-0.097)	0.064 (-0.029-0.157)
Age (y)		0.002 ^1^ (0.000-0.004)	0.005 ^3^ (0.002-0.007)	0.005 ^3^ (0.002-0.007)	0.004 ^3^ (0.002-0.007)	0.007 ^3^ (0.004-0.010)
Education	University	Ref	Ref	Ref	Ref	Ref
	Second	-0.061 (-0.137-0.015)	0.102 (-0.005-0.209)	-0.017 (-0.123-0.088)	0.000 (-0.102-0.101	0.084 (-0.043-0.211)
	Below second	-0.063 (-0.164-0.039)	0.043 (-0.101-0.187)	-0.103 (-0.246-0.039)	-0.014 (-0.149-0.121	0.233 ^2^ (0.062-0.403)
SES	ABC1	Ref	Ref	Ref	Ref	Ref
	C2DE	0.029 (-0.039-0.097)	-0.096 ^1^ (-0.191-0.000)	-0.013 (-0.107-0.082)	-0.081 (-0.171-0.010)	-0.124 ^1^ (-0.237 - -0.010)
Able to cope	Comfortable	Ref	Ref	Ref	Ref	Ref
financially	Coping	-0.104 ^2^ (-0.171 - -0.037)	0.062 (-0.033-0.157)	0.019 (-0.075-0.113)	0.008 (-0.80-0.097)	-0.052 (-0.165-0.061)
	Difficult	-0.230 ^3^ (-0.312 - -0.147)	-0.199 ^2^ (-0.318 - -0.081)	-0.020 (-0.137-0.096)	-0.140 ^1^ (-0.251 - -0.030)	-0.194 ^2^ (-0.334 - -0.053)
BMI	Healthy weight	Ref	Ref	Ref	Ref	Ref
	Overweight	0.057 (-0.020-0.134)	0.062 (-0.046-0.170)	-0.002 (-0.109-0.105)	0.014 (-0.088-0.117)	0.014 (-0.114-0.143)
	Obesity	0.118 ^1^ (0.016-0.220)	0.064 (-0.079-0.207)	0.199 ^2^ (0.059-0.339)	0.080 (-0.056-0.215)	0.019 (-0.152-0.189)
	No response	-0.151 ^2^ (-0.225 - -0.077)	0.121 ^1^ (0.017-0.225)	0.000 (-0.103-0.103)	-0.089 (-0.187-0.010)	-0.060 (-0.183-0.064)
Self-perceived	Not healthy	Ref	Ref	Ref	Ref	Ref
diet quality	Eating healthy	0.050 (-0.036-0.136)	0.213 ^2^ (0.092-0.335)	0.090 (-0.028-0.208)	0.108 (-0.007-0.222)	0.207 ^2^ (0.063-0.352)
Self-perceived	Not active	Ref	Ref	Ref	Ref	Ref
physical activity	Active	0.081 ^1^ (0.003-0.159)	0.133 ^1^ (0.024-0.242)	0.116 ^1^ (0.009-0.224)	0.107 ^1^ (0.004-0.210)	0.152 ^1^ (0.022-0.281)
Self-perceived	Not good	Ref	Ref	Ref	Ref	Ref
overall health	(Very) good	-0.015 (-0.093-0.063)	-0.031 (-0.143-0.082)	0.116 ^1^ (0.006-0.226)	0.089 (-0.015-0.193)	-0.102 (-0.236-0.032)
Health beliefs subscale		0.097 ^3^ (0.086-0.109)	0.059 ^3^ (0.043-0.075)	0.044 ^3^ (0.029-0.060)	0.075 ^3^ (0.060-0.090)	0.093 ^3^ (0.074-0.112)
Social - aesthetic beliefs subscale		-0.013 ^1^ (-0.024 - -0.002)	0.043 ^3^ (0.028-0.059)	0.005 (-0.010 – 0.020)	0.000 (-0.015-0.015)	-0.022 ^1^ (-0.040 - -0.003)
Cost subscale		-0.005 (-0.013-0.003)	-0.011 (-0.021-0.000)	-0.023 ^3^ (-0.034 - -0.012)	-0.017 ^2^ (-0.027 - -0.006)	-0.026 ^3^ (-0.039 - -0.013)
Experience of	No	Ref	Ref	*	Ref	Ref
weight stigma	Yes	-0.086 (-0.015-0.186)	-0.225 ^2^ (0.083-0.367)		0.023 (-0.111-0.157)	-0.136 (-0.305-0.032)

*Abbreviations*: *SES* socioeconomic status, *BMI* body mass index

^2^ *P* value ≤ 0.05; ^3^ *P* value ≤ 0.01; ^4^ *P* value ≤0.001

^1^* ‘Hard’ policies are laws and regulations that can limit choice and introduce financial incentives, subsidies and taxes relating to the adoption of healthy diet and physical activity behaviours *[35]

* Not included in the final regression model, as P value in the univariable model was >0.1

## Discussion

The present study analysed data from a randomly selected stratified sample of 1049 adults aged 18 + years old from the island of Ireland and explored how socio-demographic factors, anthropometrics, health behaviours, obesity-related experiences and beliefs (biopsychosocial characteristics) may influence individuals’ acceptability of policies intended to prevent or reduce obesity-related risk. Using data reduction techniques, 33 policies were clustered into five coherent categories. The most salient are discussed below.

In our sample, women had a more positive attitude towards certain initiatives aimed at reducing overweight and obesity compared to men. They specifically reported higher levels of agreement with: policies that enabled healthier environments and communities; intrusive policies such as taxation; and policies that allowed for more resources, particularly for vulnerable populations. No gender differences were observed for informational policies and advertising restrictions. Prior studies have similarly found greater acceptability of obesity-related interventions among women when compared to men [19, 21, 36, 37]. While reasons for this trend remain underexplored, there are indications from alcohol- and smoking-focused studies that women are more likely to engage in health-impairing behaviours and this may influence acceptability of initiatives to restrict such behaviours, which may also be the case in relation to obesity-focused policies here [38].

Additionally, evidence from high income countries suggests that, compared to men, women tend to have better public health literacy, i.e., better ability to evaluate health information from various sources [39, 40], which in turn may enhance their ability to value policies that aim to improve health behaviours, such as diet and physical activity.

Present findings also demonstrate that older individuals showed greater agreement with obesity policies and that was evident across all types of policies. Previous cross-sectional analyses of the demographic variability of attitudes towards governmental action against obesity in the United States (US) and Europe have also demonstrated that older respondents were more likely to endorse such policies [19, 21, 22]. The design of the existing studies, including the present analysis, is not appropriate to explain why acceptability of policies intended to prevent or reduce obesity-related risk increases with age. It can be speculated that with time people may become more aware of the physical and mental health impact of obesity. In this way, with age comes an increased desire to see more policies in place to improve public health by addressing obesity in the population, or indeed people may be more likely to have personal experience of health challenges [41].

When considering the specific types of interventions to address obesity, previous surveys conducted in Australia and the US observed that older respondents were more reluctant to pay increased taxes compared to those in the age group of 18–24 years [19, 24, 42]. In contrast, in the present analysis, age was significantly associated with an increased acceptability of ‘hard’ measures (e.g., increased taxation of foods high in sugar). When drawing these comparisons, one must consider the differences in policies implemented in those countries. For example, the sugar-sweetened drink tax has been in effect in RoI and NI (as per the whole of the UK) since 2018 [43] and, despite being primarily a tax on the industry, public attitudes, including those of older citizens, may be overall more positive compared to countries like US and Australia where a sugar tax has not been yet implemented. The link between age and individual attitudes towards obesity policies is an important consideration for policy makers and could have great potential in advancing countries’ action to support and enable the public with engagement in behaviours that positively impact upon body weight.

Financial security was directly associated with the level of agreement with most categories of obesity-related policies in the present analysis. With the exception of policies that allowed for additional resources to promote health, people who reported that they could not cope financially on their current salary were more negatively inclined towards obesity-related policies. This finding is somewhat expected considering it is well documented that fiscal policies and taxation place a greater financial burden on those on a lower income who struggle to cope financially [44, 45]. The inverse association between struggling financially and endorsing other types of interventions (i.e. policies enabling healthier environments, promotion/information-related policies and advertise restrictions) is less clear. The existing relevant literature reports mixed findings [19, 20, 36]. In this study participants reported on their subjective financial security, as opposed to their net salary. Additionally, the current analysis looked at a variety of different obesity-related policies which were grouped according to how respondents viewed them, as opposed to a specific measure targeting an obesity-related behaviour. For these two reasons our ability to compare our findings with other relevant studies is limited. It is clear however that, considering the current economic climate, the proportion of households that experience food insecurity is soaring [46, 47]. People who face challenges in securing an adequate and nutritious diet for themselves and their families are significantly more likely to be affected by obesity [48]. Policy makers need to be mindful of the association between obesity and food insecurity and future obesity-related interventions should aim at changing the food environment whilst avoiding placing additional financial burden on individuals and further exacerbating the existing inequalities in obesity prevalence [49].

In this study we observed a significant relationship between agreement with obesity policies and beliefs regarding the consequences of living with excess adiposity, particularly the beliefs around the health risks. In our sample, individuals who believed that obesity is damaging for one’s health were more likely to endorse all the types of obesity-related policies. The available evidence, as it emerges from a nationally representative online survey conducted in the US, suggests that people consider the long-term health consequences of childhood obesity to be a strong rationale for regulatory action against obesity [50]. However, less is known about people’s beliefs on the consequences of obesity throughout the life span and how they may influence public attitudes towards obesity policies. While the link between obesity health beliefs and agreement with policy action warrants further exploration, our findings highlight the importance of addressing the health consequences of living with overweight in an evidence-based, non-stigmatising manner.

Residents of NI showed greater levels of agreement with overall obesity policies when compared to those living in the RoI. Despite sharing a generally small land mass (70 km^2^), the two populations showed a different attitude towards obesity-related policies. This is a reminder that the data for this study were collected in two countries with distinct political and healthcare contexts (universal public healthcare in NI versus a substantial proportion of pay-out-of-pocket services in RoI). As seen in previous studies that explored the determinants of attitudes towards approaches against obesity, the level of acceptance of a certain measure may be influenced by political ideologies and the level of trust in the government in power [19, 21, 22], which may have been the case in the present sample. Additionally, this finding gives an insight into differences in how the public perceives obesity in these two countries. The latest data show that the prevalence of living with excess adiposity in adults and children is higher in NI (65% and 25.5% respectively) [10] compared to the RoI (56% and 24% respectively) [9, 11]. Additionally, an analysis estimating the healthcare and productivity cost of obesity on the island of Ireland concluded that the overall cost per capita in NI exceeds by far that of RoI [51]. These factorsmay have influenced NI respondents’ views.

Personal experiences of weight stigma and attitudes towards obesity policy were also explored. Reporting personal experience of weight-based discrimination was associated with lower levels of agreement with ‘‘hard’’ policies. No association with other types of policies was observed. ‘‘Hard’’ policies include public health interventions such as routine monitoring of children’s height and weight, as well as increased taxation for foods high in sugar and fat. These approaches may be perceived by some as oversimplifying weight management, as it has been argued is done in current nutritional guidelines [52]. Intrusive or ‘hard’ policies may be also seen as restricting personal choice, or even punishing and further reinforcing discrimination and stigmatising behaviours. The presence of weight bias in today’s society on the island of Ireland has been previously documented [26]. However less is known about its potential link to agreement with obesity policies. The methodology followed in this study does not allow for explaining how agreement (or disagreement) with certain restrictive obesity interventions may be influenced by personal experiences of being discriminated against due to one’s weight. However, our finding highlights that future efforts to increase public endorsement of such policies needs to first raise awareness of weight stigma and ways to avoid it.

### Strengths and limitations

Few studies have examined the relationship between attitudes towards obesity-policy interventions and demographic characteristics. This is the first study to look at the level of acceptance of a wide range of obesity-related policies (*n* = 33) (spanning multiple layers of the system, e.g. fiscal measures, regulation, environmental/social planning, education and service provision) and its link with factors that go beyond demographics and pertain to the biopsychosocial spectrum, such as anthropometrics, health behaviours and obesity-related beliefs and experiences. Strong methodological aspects of this study include a large stratified random sample and a survey which elucidated public views on specific approaches to address obesity, as opposed to broadly formulated approaches, as in previous studies.

Due to inherent challenges in assessing quality of diet, health status and level of physical activity, it was decided that in this study these measurements would be self-reported. This may have compromised the accuracy of these measurements, as people tend to over- or underestimate in the absence of a clear reference as to what constitutes good health, a high physical activity level or healthy eating behaviours. Additionally, a substantial proportion of missing data in relation to respondents’ anthropometric measurements (a third of the sample) meant that we were not able to obtain a BMI for them and consequently, accurately assess whether and how BMI is associated with public attitudes towards obesity policies. Moreover, our data collection looked at various aspects of demographic profile and health behaviours, but we are mindful of the fact that there is a plethora of contextual factors that may influence public attitudes towards obesity policy actions, which were not evaluated in the present exploration, such as trust (or lack thereof) in the food industry or government’s agenda.

This survey yielded a large dataset and PCA was chosen as the appropriate approach to data reduction and identification of patterns [30], similarly to previous studies that explored potential determinants of agreement with obesity policy [20–22, 24]. The authors are mindful that this was an exploratory, data-driven statistical approach and one that does not rely on pre-existing theoretical constructs and hypotheses and therefore, its generalisability to other populations is limited.

One final methodological consideration is the decision to exclude four policies and include 33 of them in the regression analysis. This decision was based on current literature suggesting that in big samples (such as this one), items with low correlation coefficient (< 0.4) be dropped; particularly when there are several items/policies with adequate to strong correlation coefficients (> 0.5) to each factor, as it happens with the current exploration [32, 33]. This decision was also taken whilst considering the integrity of our data and the absence of significant correlation between these four policies and the extracted factors (i.e., policy categories), which reflects how our participants have perceived them and subsequently reported agreement/disagreement with them.

## Conclusions

This survey demonstrates that there is great variation in public attitudes towards obesity policies based on individuals’ demographic profile, obesity-related beliefs and personal experiences of weight-based discrimination across a broad variety of policy measures targeting varying levels of the wider system. Our findings suggest that age, gender, financial security, country of residence, beliefs around the health consequences of obesity and experiences of weight stigma may be linked to how the public views different policies to address obesity, such as healthy school meals, nutrition education and more exercise facilities. Policy makers may consider these findings when aiming to secure agreement with specific preventative approaches and their advocacy efforts should be targeted to the subgroups that demonstrate lower levels of agreement (men, younger age groups and those finding difficult to cope financially) and finding solutions to meet their needs. Whilst these prevention and obesity-risk reduction policy approaches are of major importance for the health of the population, there remains a need for adequate treatment and management approaches for the significant proportions of the population who are living with obesity and related health complications.

## Supplementary Information

Supplementary Material 1.

Supplementary Material 2.

Supplementary Material 3.

Supplementary Material 4.

## Data Availability

The datasets used and/or analysed during the current study are available from the corresponding author on reasonable request.

## Abbreviations

RoI: Republic of Ireland
NI: Northern Ireland
UK: United Kingdom
WSA: Whole-system approach
NHS: National Health System
PCA: Principal Component Analysis
BMI: Body mass index
UG: Undergraduate
PG: Postgraduate
NVQ: National vocational qualification
SES: Socioeconomic status
US: United States
