# Supplementary material for: Public attitudes towards obesity policies on the island of Ireland; exploring the relationship with biopsychosocial characteristics
Source: BMC Public Health. 2025 Oct 9;25:3436. doi: 10.1186/s12889-025-24216-8 (PMC12512851; doi:10.1186/s12889-025-24216-8)
Supplement: Supplementary file 1 — Supplementary Material 1. [file 12889_2025_24216_MOESM1_ESM.pdf]

Section A: Health and Wellbeing

The following questions are about obesity on the island of Ireland, and ways that it can be managed. Through these questions, we want to find out opinions held by the general public. Your responses are very important to us.

The first questions are about your health and wellbeing. They are about how you perceive yourself and what you do in your day to day life. They in no way make a judgement about yourself

A1. Overall would you say that what you usually eat is..? **(Single code)** SHOWCARD B

Very healthy	1
Quite healthy	2
Not very healthy	3
Very unhealthy	4

A2. Overall how would you describe your physical activity level (where you get out of breath)? SHOWCARD C

Very physically active	1
Quite physically active	2
Not very physically active	3
Not at all physically active	4

A3. How easy or difficult would it be to make improvements to the way you eat? **(Single code)** SHOWCARD C

Very easy	1
Quite easy	2
Quite difficult	3
Very difficult	4
No changes necessary	5

A4. How easy or difficult would it be to make improvements to how much physical exercise (out of breath) you get? **(Single code)** SHOWCARD E

Very easy	1
Quite easy	2
Quite difficult	3
Very difficult	4
No changes necessary	5

The following parts of the survey may contain concepts and questions that some people may find upsetting. There are questions relating to stigma, discrimination and eating disorders. If you have any concerns during the survey, please let the me (interviewer) know. If you have had past experience with eating disorders and need help, we can provide contact details for help services.

INTERVIEWER NOTE: READ OUT IF ANY DISTRESS EXHIBITED BY RESPONDENT: *In Ireland, you can also get help from Body Whys on 1890 200 444 and through their online support group. In Northern Ireland, you can get help from the Eating Disorder Association on 028 9023 5959, or through their website.*

We would like to highlight that these views aren't views necessarily held by the researchers. These questions are meant to get your opinions about obesity as accurately as possible. However, it is important to make sure we capture all opinions. The opinions may not be held by you but might be by other people.

Section B: Views about Obesity

In this section, we want to know what you think about obesity in general.

B1. Here are a series of explanations people in the general public give about why people are overweight or obese. We use the terms such as "people with obesity". These may not sound grammatically correct sometimes. We treat obesity as a condition, similar to diabetes. In response to each statement, could you please tell us how much you agree with the view presented, where (1) is strongly disagree and (5) is strongly agree. Please choose the number that best represents your view. **(Single code for each statement)**

This first group of questions are about factors that might lead to people becoming overweight or developing obesity on the island of Ireland. SHOWCARD F

Strongly disagree	1
Disagree	2
Neither agree nor disagree	3
Agree	4
Strongly agree	5
Don't know	6
Refused	7

1	Being overweight is something you inherit from your parents.
2	There is too much unhealthy and fatty food in restaurants, supermarkets and convenience stores. (shops)
3	Most weight-loss diets are not very effective
4	People lack the willpower to diet or exercise regularly.
5	People become overweight because they spend too much time driving/watching television/on the internet.
6	People become overweight because they don't have time to prepare healthy meals.
7	People become overweight because they are simply born that way
8	Most people who become overweight don't view their weight as a problem.
9	People become overweight because they are unable to buy healthy food close to their home.
10	People become overweight because there are too many snack foods readily available in workplaces, shops and homes.
11	People become overweight because healthy foods are too expensive.
12	People who become overweight eat whatever they want.
13	People become overweight because they don't have time to exercise.
14	People who eat too much unhealthy food do so because it costs much less than healthy food.
15	Most people in society can't afford to eat healthy foods and exercise regularly.
16	People become overweight because they lack information about healthy eating and/or health risks of excess weight.
17	People become overweight because they value more immediate satisfaction compared to future health risks.

B2. This next set of questions is about views that people may have about obesity in general. Like the last section, please say how much you agree with these statements. **(Single code for each statement)** SHOWCARD G

1	People have to deny themselves a great deal to avoid obesity.
2	Maintaining a healthy bodyweight is expensive.
3	People should maintain a healthy weight for optimal health.
4	Maintaining a healthy bodyweight is boring.
5	People with obesity would be treated better if they lost weight.
6	Maintaining a healthy bodyweight takes a lot of effort.
7	A person with a healthy bodyweight can lead a more active life.
8	Maintaining a healthy bodyweight makes life less fun.
9	People with overweight or obesity are considered less attractive.
10	People with obesity need more medical care.
11	People with obesity are embarrassed by the way they look.
12	Losing weight would greatly improve the health of people living with obesity.
13	People with obesity would have a better social life if they lost weight.
14	A person who avoids obesity has a restricted lifestyle.
15	People with a healthy bodyweight are taken more seriously.
16	Getting older causes people to become overweight.
17	People can be overweight and still be healthy
18	People who have obesity live shorter lives

Section C: Views of policy interventions

The next section refers to ways the government might prevent and reduce obesity in the population, which we will refer to as "policy". The following questions ask about responsibility for obesity in society, how you think about government policy, and how you feel about different policy ideas. There are no right and wrong answers.

C1. People in our society often debate about how to balance individual responsibility against the responsibilities of the government and society. Please indicate how strongly you agree or disagree with each of these statements where (1) represents strongly disagree and (5) represents strongly agree. **(Single code for each statement)** SHOWCARD J

Strongly disagree	1
Disagree	2
Neither agree nor disagree	3
Agree	4
Strongly agree	5
Don't know	6

1	The government interferes far too much in our everyday lives.
2	Sometimes the government needs to make laws that keep people from hurting themselves.
3	The government should stop telling people how to live their lives.
4	It's not the governments business to try and protect people from themselves.
5	The government should do more to advance society's goals even if that means limiting the freedom and choices of individuals.
6	Governments should put limits on the choices individuals can make so they don't get in the way of what's good for society.

C2. To what extent do you either agree or disagree with each of the following statements about government interventions were (1) is strongly disagree and (5) is strongly agree? **(Single code for each statement)** SHOWCARD K

1	The government should impose taxes on unhealthy foods and use the proceeds to promote healthier eating.
2	The government should subsidise (make cheaper) fruit and vegetables to promote healthier eating.
3	The government should provide vouchers to low-income families to buy healthy foods at reduced prices.
4	Vending machines selling unhealthy food should be banned from our schools (including secondary schools).
5	The government should make sure that primary school meals meet a healthy standard of nutrition
6	The government should make sure that secondary school meals meet a healthy standard of nutrition
7	The government should make sure that meals sold or provided at workplaces meet a healthy standard of nutrition
8	The government should make sure that meals available in hospitals to staff and visitors meet a healthy standard of nutrition
9	The government should work with the food companies to improve the nutritional content of processed foods (e.g. less salt or fats).

C3. **(Single code for each statement)** SHOWCARD L

1	The government should impose limits on certain ingredients (e.g. salt or fats) on food companies to improve the nutritional content of processed foods.
2	TV-stations should give free air-time to governmental campaigns that promote healthier eating.
3	There should be public measures like free home delivery to support easier access to healthy foods for the elderly and those with lower incomes.
4	VAT (value added tax) rates should be lower for healthy foods and higher for unhealthy foods.
5	The government should ban advertising for unhealthy food that is aimed at children.
6	The government should ban advertising for unhealthy food that is aimed at adults.
7	The government should spend money on effective campaigns informing people about the risks of unhealthy eating.
8	Education to promote healthy eating should be provided in all schools.
9	Practical education in food preparation should be taught in all schools
10	The government should subsidise businesses which provide programmes to support their employees in healthy eating
11	All foods should be required to carry understandable labels with calorie and nutrient information.
12	All restaurants should be required to provide calorie and nutrient information on menus.
13	The food industry should help pay for governmental campaigns that promote healthy eating.

C4. **(Single code for each statement)** SHOWCARD M

1	The government should reward companies for healthy food innovations.
2	Children should have to participate in a minimum of 30 minutes exercise a day while at school.
3	There should be planning regulations to restrict the development of fast food outlets in areas nearby to schools.
4	The government should provide resources to improve exercise and playground facilities
5	The government should provide resources to encourage women to breastfeed
6	There should be planning regulations to restrict the development of certain food outlets (selling foods high in saturated fats) in towns and cities.
7	There should be a ban on sales promotion and special offers on unhealthy foods.
8	Portion sizes in restaurants and fast food shops should be restricted
9	There should be a tax incentive to encourage sports participation, with a tax break for the purchase of relevant sports equipment.
10	There should be an additional health charge for those presenting with obesity. **(X4 EQ 2 ONLY)**
11	There should be health insurance price reductions for those of healthy weight. **(X4 EQ 2 ONLY)**

C5. Please say if you agree or disagree with each of the following statements: **(Single code for each statement)** SHOWCARD N

1	The government should extend the sugar sweetened drinks tax to include all sugary foods to promote healthier eating
2	The government should ban companies that make unhealthy foods and drinks from sponsoring children's organisations, children's events and children's sporting teams
3	The government should restrict advertising for unhealthy food in public spaces (e.g. bus stops, trains stations, hospitals, roadside)
4	The government should restrict advertising for unhealthy food that is aimed at children on the internet (e.g. games, apps, social media)
5	Children's height and weight should be routinely measured to monitor rates of growth in the population
6	The government should try and make towns and cities so that people are encouraged to be more active and healthier (such as bike lanes, parks, pedestrian areas)

Section E. Profile information

Finally, we would like some background information about you. This is a critical part of our analysis so we can sure we take into consideration people of all backgrounds. The questions are in no way intended to make a judgement.

E1. Are you...? **(Single Code)**

Male	Female	Non Binary	Prefer not to say
1	2	3	4

E2. What was your age on your last birthday? INTERVIEWER RECORD AGE:

E3. How would you rate your overall health, all things considered? **(Single Code) SHOWCARD S**

Very bad	1
Bad	2
Fair	3
Good	4
Very good	5

E4. How tall are you? **(Single Code)**

Respondent answered in feet and inches	1
Respondent answered in meters and centimetres	2
Refused	3

E6. What is your weight? **(Single Code)**

Respondent answered in stones and pounds	1
Respondent answered in kilograms	2
Refused	3

E8. Have you ever had a medical treatment for weight issues (such as medication, counselling or surgery)? **(Single code)**

Yes	1
No	2

E9. What is your marital status? **(Single code) SHOWCARD T**

Single (never married)	1
Married	2
Civil Partnership	3
Divorced	4
Domestic partnership (cohabiting but not married)	5
Separated	6
Widowed	7
Prefer not to say / refused	8

E10. Are you currently in full-time education? **(Single code)**

Yes	1
No	2

ASK IF X1 EQ 1

E11a. Which of the following best describes your level of education? [IF STILL STUDYING: Which level best describes your level of education you obtained until now? **(Single Code)** SHOWCARD U

Some primary (not complete)	1
Primary or equivalent	2
GCSE	3
A 'Level	4
Apprenticeship/ Trade Certificate/ training	5
Diploma/Certificate	6
Primary degree/ Nursing Qualification (B.Sc., B.A., etc)	7
Postgraduate (M.A., Ph.D., etc)	8
Other (please specify)	9

ASK IF X1 EQ 2

E11b. Which of the following best describes your level of education? [IF STILL STUDYING: Which level best describes your level of education you obtained until now? **(Single Code)** SHOWCARD V

Some primary (not complete)	1
Primary or equivalent	2
Intermediate/ Junior/ Group Certificate or equivalent	3
Leaving certificate or equivalent	4
Apprenticeship/ Trade Certificate/ FAS training	5
Diploma/Certificate	6
Primary degree/ Nursing Qualification (B.Sc., B.A., etc)	7
Postgraduate (M.A., Ph.D., etc)	8
Other (please specify)	9

E12. What is or was the occupation of the highest income earner in your household?
INTERVIEWER RECORD OCCUPATION

E13. INTERVIEWER CODE SOCIAL CLASS OF CHIEF INCOME EARNER: ? **(Single Code)**

A	1
B	2
C1	3
C2	4
D	5
E	6
F (50+)	7
F (LESS THAN 50)	8

E14. How would you describe your nationality? **(Single Code)** SHOWCARD W

British	1
Irish	2
Northern Irish	3
English	4
Scottish	5
Welsh	6
Polish	7
Lithuanian	8
Romanian	9
Brazilian	10
Latvian	11
Spanish	12
Other (please specify) [pop up box]	13

E15. Which of the descriptions comes closest to how you feel about your household's income nowadays? **(Single code)** SHOWCARD X

Living very comfortably on present income	1
Living comfortably on present income	2
Coping on present income	3
Finding it difficult on present income	4
Finding it very difficult on present income	5
Don't know / prefer not to say	6

E16. How many children under the age of 18 live in your household? **(Insert number if none type 0)**

ASK IF X1 EQ 2

E17. Please say if you have each of the following..? **(Code all that apply)** SHOWCARD Y

Full Medical Card or equivalent	1
Visit Card	2
Private medical insurance cover	3
I have none of the above	4

ASK IF X1 EQ 1

E18. Do you have private medical insurance? **(Single Code)**

Yes	1
No	2

THANK AND CLOSE
