# Supplementary material for: Public attitudes towards obesity policies on the island of Ireland; exploring the relationship with biopsychosocial characteristics
Source: BMC Public Health. 2025 Oct 9;25:3436. doi: 10.1186/s12889-025-24216-8 (PMC12512851; doi:10.1186/s12889-025-24216-8)
Supplement: Supplementary file 2 — Supplementary Material 2. [file 12889_2025_24216_MOESM2_ESM.docx]

Supplementary Table 1. Support for 39 obesity policies in the Republic of Ireland and Northern Ireland, as calculated by summing the proportion of respondents who answered “Agree” or “Strongly agree”. The policies are presented in descending order based on percentage of agreement (N=1049)

| **Policy** | **Overall Agreement (%)** |
| --- | --- |
| Practical education in food preparation should be taught in all schools. | 91.7 |
| Education to promote healthy eating should be provided in all schools. | 91.4 |
| The Government should make sure that meals available in hospitals to staff and visitors meet a healthy standard of nutrition. | 90.9 |
| The Government should make sure that secondary school meals meet a healthy standard of nutrition. | 90.6 |
| The Government should provide resources to improve exercise and playground facilities. | 90.1 |
| The Government should make sure that meals sold or provided at workplaces meet a healthy standard of nutrition. | 88.8 |
| The Government should work with the food companies to improve the nutritional content of processed foods (less salt or fats). | 88.7 |
| Children should have to participate in a minimum of 30-minute exercise a day while at school. | 85.3 |
| The Government should spend money on effective campaigns informing people about the risks of unhealthy eating. | 83.7 |
| The Government should subsidise (make cheaper) fruit and vegetables to promote healthier eating. | 83.5 |
| All foods should be required to carry labels with calorie and nutrient information. | 83.0 |
| The Government should try to make towns and cities such that people are encouraged to be more active and healthier (such as bike lanes, parks, pedestrian areas). | 81.0 |
| The Government should restrict advertising for unhealthy food that is aimed at children on the Internet. | 80.7 |
| VAT (value added tax) rates should be lower for healthy foods and higher for unhealthy foods. | 80.3 |
| The Government should ban advertising for unhealthy food that is aimed at children. | 80.2 |
| The Government should impose limits on certain ingredients (for example salt or fats) on food companies to improve the nutritional content of processed foods. | 78.9 |
| All restaurants should be required to provide calorie and nutrient information in menus. | 78.0 |
| Vending machines selling unhealthy food should be banned from our schools (including secondary schools). | 77.8 |
| There should be public measures like free home delivery to support easier access to healthy foods for the elderly and those with lower incomes. | 77.6 |
| **Policy** | **Overall Agreement (%)** |
| The Government should provide vouchers to low-income families to buy healthy foods at reduced prices. | 75.9 |
| The Government should reward companies for healthy food innovations. | 75.1 |
| There should be a tax incentive to encourage sports participation, with a tax break for the purchase of sports equipment. | 74.4 |
| The Government should subsidise businesses which provide programmes to support their employees in healthy eating. | 73.7 |
| The food industry should help pay for governmental campaigns that promote healthy eating. | 72.9 |
| The Government should provide resources to encourage women to breastfeed. | 72.7 |
| Television stations should give free air-time to governmental campaigns that promote healthier eating. | 70.1 |
| The Government should ban advertising for unhealthy foods that is aimed at adults. | 69.4 |
| The Government should restrict advertising for unhealthy food in public spaces (for examples bus stops, trains stations, hospitals, roadside). | 68.8 |
| The Government should extend the Sugar Sweetened Drinks Tax to include all sugary foods to promote healthier eating. | 68.3 |
| The Government should impose taxes on unhealthy foods and use the proceeds to promote healthier eating. | 67.5 |
| There should be planning regulations to restrict the development of fast-food outlets in areas near to schools. | 66.5 |
| The Government should ban companies that make unhealthy foods and drinks from sponsoring children’s organisations, children’s events and children’s sporting teams. | 65.5 |
| Children’s height and weight should be routinely measured to monitor rates of growth in the population. | 63.3 |
| There should be a ban on sales promotion and special offers on unhealthy foods. | 61.4 |
| There should be health insurance price reductions for those of healthy weight. | 60.5 |
| There should be planning regulations to restrict the development of certain food outlets (selling foods high in saturated fats) in towns and cities. | 60.4 |
| Portion sizes in restaurants and fast-food shops should be restricted. | 47.3 |
| There should be an additional health charge for those presenting with obesity. | 39.6 |
| Practical education in food preparation should be taught in all schools. | 91.7 |
