# Supplementary material for: Public attitudes towards obesity policies on the island of Ireland; exploring the relationship with biopsychosocial characteristics
Source: BMC Public Health. 2025 Oct 9;25:3436. doi: 10.1186/s12889-025-24216-8 (PMC12512851; doi:10.1186/s12889-025-24216-8)
Supplement: Supplementary file 4 — Supplementary Material 4. [file 12889_2025_24216_MOESM4_ESM.docx]

Table 3. Tucker’s congruence coefficient matrix following a split-sample replication of the Principal Component Analysis. All five components show excellent replication (congruence coefficient > 0.90).

| **Second split** | **First split** | | | | | |
| --- | --- | --- | --- | --- | --- | --- |
|  |  | Component 2 | Component 4 | Component 5 | Component 1 | Component 3 |
|  | Component 2 | **0.98** | 0.12 | 0.17 | 0.18 | 0.21 |
|  | Component 4 | 0.11 | **0.96** | 0.11 | 0.37 | 0.20 |
|  | Component 5 | 0.25 | 0.24 | **0.96** | 0.24 | 0.25 |
|  | Component 1 | 0.12 | 0.15 | 0.28 | **0.93** | 0.09 |
|  | Component 3 | 0.07 | 0.07 | 0.17 | -0.03 | **0.92** |

*Table 4. Results of Horn's Parallel Analysis for component retention (100 iterations, using the mean estimate). Adjusted eigenvalues > 1 indicate components to retain.*

| Component | Adjusted Eigenvalue | Unadjusted Eigenvalue | Estimated Bias |
| --- | --- | --- | --- |
| 1 | 12.340114 | 12.686849 | 0.346735 |
| 2 | 2.806104 | 3.107233 | 0.301129 |
| 3 | 1.360426 | 1.632519 | 0.272093 |
| 4 | 1.342373 | 1.585147 | 0.242774 |
| 5 | 1.185820 | 1.406335 | 0.220515 |
| 6 | 0.852112 | 1.047469 | 0.195356 |
| 7 | 0.697254 | 0.869297 | 0.172042 |
| 8 | 0.673687 | 0.825923 | 0.152235 |
| 9 | 0.636541 | 0.770110 | 0.133568 |
| 10 | 0.603023 | 0.715967 | 0.112944 |
| 11 | 0.533530 | 0.627020 | 0.093489 |
| 12 | 0.543315 | 0.616991 | 0.073675 |
| 13 | 0.501260 | 0.558922 | 0.057661 |
| 14 | 0.483770 | 0.523791 | 0.040021 |
| 15 | 0.470148 | 0.493778 | 0.023630 |
| 16 | 0.464140 | 0.470842 | 0.006701 |
| 17 | 0.464140 | 0.470842 | 0.006701 |
| 18 | 0.440223 | 0.413630 | -0.02659 |
| 19 | 0.453435 | 0.411226 | -0.04220 |
| 20 | 0.453214 | 0.393421 | -0.05979 |
| 21 | 0.452944 | 0.378562 | -0.07438 |
| 22 | 0.461395 | 0.371356 | -0.09003 |
| 23 | 0.466120 | 0.359608 | -0.10651 |
| 24 | 0.453786 | 0.331293 | -0.12249 |
| 25 | 0.450387 | 0.311614 | -0.13877 |
| 26 | 0.443931 | 0.286218 | -0.15771 |
| 27 | 0.416617 | 0.242412 | -0.17420 |
| 28 | 0.415526 | 0.224050 | -0.19147 |
| 29 | 0.427803 | 0.216800 | -0.21100 |
| 30 | 0.429124 | 0.201079 | -0.22804 |
| 31 | 0.433743 | 0.186570 | -0.24717 |
| 32 | 0.429660 | 0.160584 | -0.26907 |
| 33 | 0.426843 | 0.130918 | -0.29592 |
